# Response Clustering of Reverse-Keyed, Adversely Framed Items in the Human Rights-Based Well-Being Checklist: An Exploratory Network Analysis

**DOI:** 10.7759/cureus.117079

**Published:** 2026-09-28

**Authors:** Fumie Higashira, Takashi Izutsu, Shodai Sunagozaka, Satoshi Iiyama, Atsuro Tsutsumi

**Affiliations:** 1 Graduate School of Human and Socioenvironmental Studies, Kanazawa University, Kanazawa, JPN; 2 Graduate School of Agricultural and Life Sciences, The University of Tokyo, Tokyo, JPN; 3 Faculty of Arts and Science, Kyushu University, Fukuoka, JPN; 4 Graduate School of Medicine, The University of Tokyo, Tokyo, JPN; 5 Institute of Transdisciplinary Sciences for Innovation, Kanazawa University, Kanazawa, JPN

**Keywords:** convention on the rights of persons with disabilities (crpd), crpd, disability, human rights-based well-being checklist (hrwc), network analysis, philippines, reverse-keyed items

## Abstract

Introduction: The Convention on the Rights of Persons with Disabilities (CRPD) enumerates a broad, heterogeneous set of rights and requires their monitoring. The Human Rights-Based Well-Being Checklist (HRWC) is a 39-item instrument aligned with the CRPD; it covers this heterogeneous content, and no subscales were defined at its development. Using exploratory network analysis, we examined the response structure of the HRWC to clarify its descriptive interpretation and to assess whether empirically detected item communities were sufficiently stable to justify subscale construction or item removal. The focal case was a set of eight items that are reverse-keyed in the scoring scheme and framed in terms of adverse rights-related experiences (Items 1-4, 12, 14, 17, and 18). This set was defined by the scoring scheme independently of the network results; the decision to focus on it was post hoc.

Methods: This secondary analysis used registry data from adults with disabilities in Muntinlupa, Philippines (N = 1,014). The HRWC is a self-report instrument whose items describe day-to-day situations corresponding to CRPD rights; each item is scored 0-4, giving a possible total of 0-156. Regularized partial-correlation networks, in which each edge represents the association between two items after accounting for all the other items, were estimated under two parallel specifications: rank-based Spearman correlations, which use only the rank order of the responses, and polychoric correlations, which treat each response as a coarsened observation of an underlying continuous variable. Item communities, that is, groups of items more densely interconnected with one another than with the rest of the network, were detected with the Walktrap algorithm, and their reproducibility was examined in 1,000 bootstrap resamples per specification.

Results: Under Spearman estimation, the eight items formed one community containing no other items (27 of 28 possible within-set edges positive and nonzero; one absent), were retained across algorithms, regularization settings, and the nonparanormal network, and were reproduced in 885 of the 1,000 resamples (88.5%). Under polychoric estimation, the Walktrap point estimate divided the eight items along a shared stem (Items 1-4 versus 12, 14, 17, 18), although three alternative detection algorithms recovered that single community; the same eight-item-only community recurred under Walktrap in 333 of the 1,000 resamples (33.3%). Whether the eight items formed one community or two subgroups, thus, varied with the correlation method, the detection algorithm, and the resample.

Conclusions: Item-level profiles and the existing unweighted total serve different descriptive functions. Item-level profiles preserve information on the distinct CRPD-related conditions represented by individual items, whereas the unweighted total can provide a concise summary across all 39 responses. Substantive content could not be separated from keying, shared stems, item position, or response distributions, and the findings provide no empirical support from these analyses for constructing subscales or removing items on the basis of the observed network communities.

## Introduction

The Convention on the Rights of Persons with Disabilities (CRPD) enumerates a broad, heterogeneous set of rights and requires their monitoring [1]. The Human Rights-Based Well-Being Checklist (HRWC) is a 39-item questionnaire developed with United Nations bodies and disability experts to summarize reported individual-level experiences related to CRPD rights [2-3]. Its items describe day-to-day situations corresponding to the rights in the CRPD; each maps onto one or more articles (Appendix A; mappings compiled with the instrument developers, who are coauthors), and no subscales were defined at development. In the present report, well-being refers to the rights-related conditions of everyday life described in the CRPD articles, as reported by the respondent [2]. The instrument covers heterogeneous CRPD-related content and has no predefined reflective subscales. For this analysis, we treat it as a content-coverage instrument with index-like or composite characteristics [4-5]. This interpretation was adopted for the present analysis; it was not specified during instrument development. Interitem covariance may reflect both the substantive co-occurrence of rights-related conditions and shared item or response features; we do not treat it as a validity criterion by itself, and the present analysis does not impose or test a reflective measurement structure. Previous studies reported total-score internal consistency [2]; its internal response structure has not been examined.

Eight HRWC items warrant particular attention because several features coincide. These items are reverse-keyed and framed in terms of adverse rights-related experiences: discrimination, threats to life, obstruction of legal decision-making, unlawful detention, and physical or sexual violence (hereafter, adversely framed items; Items 1-4, 12, 14, 17, and 18); most are not simple grammatical reversals [2]. They carry a negative valence, four share a near-identical stem, and their response distributions differ from those of the positively worded items. This report examines clustering associated with these shared features. Visually distinct communities may invite subscale construction, item removal, or rescoring. Such decisions may be inappropriate when grouped items carry noninterchangeable content and composition varies across specifications. We, therefore, examined whether the observed communities were sufficiently stable to justify changes to scoring or item content.

Groupings of reverse-keyed items have been reported repeatedly in factor-analytic and related psychometric studies of instruments whose substantive content is otherwise heterogeneous. Such groupings have been attributed to careless responding [6], to wording effects that are not resolved by modeling them explicitly [7], and to the influence of item keying on the recovered factor structure [8]. These reports concern reflective scales, in which a keying-related grouping is generally treated as a nuisance to be modeled away or removed. Whether the same treatment is appropriate for an instrument that is not reflective and in which the grouped items describe distinct and noninterchangeable rights-related conditions has not been examined.

In registry-based surveys and local disability-service research, item-level summaries may be used to describe heterogeneous rights-related needs, so how responses cluster bears on how such summaries are interpreted. The eight items described above are likely targets of such decisions.

The aim of the present study was to examine how HRWC item responses are organized in a regularized partial-correlation network and whether the resulting communities were sufficiently stable across defensible analytic specifications to support subscale construction, item removal, or changes to the existing scoring structure. In practical terms, the question addressed is whether the eight adversely framed items should be grouped, kept separate, or removed. The eight-item set was defined independently of the network results, although the decision to focus on it followed exploratory inspection of the estimated networks.

## Materials and methods

Participants and procedure

This study is a secondary methodological analysis of data from a broader survey program on disability, rights, and well-being; an earlier publication from that program compared traumatic experiences and human rights violations across disability groups [3]. A cross-sectional, registry-based survey was conducted in Muntinlupa City, Metro Manila, Philippines, between January 2019 and January 2020 [3]. Participants were randomly selected from the municipal registry of persons with disabilities, in which registration was based on clinicians' judgment; health workers employed by the city government, with clinical expertise and sensitivity to disability-related issues, visited households to collect data. Of 3,000 registrants initially approached, 1,024 completed the survey (34.1%). Recorded reasons for nonparticipation were refusal, absence during the household visit, and inability to answer the questions owing to the nature of their disabilities. Counts by reason were unavailable. The questionnaires were distributed at the household visit, with the health workers providing verbal confirmation of the items and responses as needed. HRWC items were presented bilingually in Filipino (Tagalog) and English in the printed order of the instrument, and in both languages, Items 1-4 share a near-identical stem.

Study population and eligibility

Inclusion criteria were that the respondent was among the registrants approached as described above and that the questionnaire was completed. One exclusion criterion was applied in the present analysis and was not used in the earlier publication [3]: the present analysis was restricted to records with a recorded age of 18 years or over. Ten records carried a recorded age below 18 years and were, therefore, excluded. Participant flow was, therefore, as follows: 3,000 registrants approached, 1,024 completed questionnaires (34.1%), 10 records excluded on this criterion, and a primary analysis sample of 1,014. No other exclusion criterion was applied: no record was dropped for missing data, because there were no missing responses on the 39 HRWC items, and no respondent was excluded on the basis of registered disability type, sex or gender, employment status, or any HRWC response. The 1,014 eligible records were therefore analyzed as a single sample without stratification.

Sample size determination

No a priori sample size or power calculation was performed for the present analysis; the sample size determination of the survey from which these data come was based on a comparison of HRWC total scores between disability groups and is reported in the earlier publication [3]. As this is a secondary analysis, the number of observations was fixed by the number of completed questionnaires, and all 1,014 eligible records were analyzed. Because the analyses are descriptive, that number determines the precision with which the 741 possible item pairs are estimated rather than the power of any test. The adequacy of the available sample for the planned analyses was therefore addressed empirically rather than by an a priori rule. All 741 item pairs were estimated from the same 1,014 observations, and the stability of the estimated networks and of the Walktrap communities was not assumed but examined directly, in 1,000 nonparametric bootstrap resamples per specification, with the reproduction rates reported in the Results section. Where a network or community partition proved unstable under this procedure, that instability is reported as a finding rather than resolved by a modeling choice.

Measures

The HRWC is a 39-item self-report instrument capturing respondents' reported rights-related conditions, scored on a five-point Likert scale (0-4) with a possible total score of 0-156; in the present survey it was completed on the distributed questionnaire at the household visit, with verbal confirmation by the health workers as needed. The reverse-keyed, adversely framed items (1-4, 12, 14, 17, 18) were rescored so that higher values indicate more favorable reported rights-related conditions. These eight items are defined by the scoring scheme of the instrument and are therefore identifiable before any network is estimated. Their content covers inequality and discrimination on the grounds of sex or gender, age, disability, and race or ethnicity (Items 1-4), and threats to life, obstruction of legal decision-making, unlawful detention, and physical or sexual violence (Items 12, 14, 17, and 18). Two of the authors (T.I. and A.T.) were involved in the original development of the HRWC and contributed to the CRPD-article mapping presented in Appendix A; the network analyses reported here were executed by the remaining authors.

Statistical analysis

No analysis plan was preregistered; all analyses were exploratory. No numerical stability threshold was prespecified; stability was judged by whether the same composition recurred across these specifications and across resamples. The eight-item set was defined from the scoring scheme, not from the network results; the decision to focus on it, however, followed exploratory inspection of the estimated networks and is therefore post hoc.

Network estimation

Regularized partial-correlation networks were estimated with graphical lasso [9] and extended-Bayesian-information-criterion model selection (EBICglasso; γ = 0.50) [10] under two parallel principal correlation specifications [11]: rank-based Spearman correlations and ordinal polychoric correlations [12], both computed after reverse coding. The two specifications differ in what they assume about the response scale. Spearman correlations use only the rank order of the observed responses, whereas polychoric correlations model each item as a coarsened observation of an underlying continuous variable. Both are defensible for five-point items, and neither was treated as the correct specification; they were therefore estimated in parallel and reported side by side throughout.

Community detection

A community is a group of items that are more densely interconnected with one another than with the rest of the network; communities in this report are descriptive groupings and are not subscales. The communities were detected with the Walktrap [13] algorithm (four steps; absolute partial-correlation weights). Walktrap was adopted as the principal detection method before interpretation of the community results because it identifies locally cohesive groups through short random walks; given the exploratory design, alternative algorithms were examined without treating Walktrap as the gold standard. Because absolute weights discard edge signs, sign composition was inspected directly. Three further community-detection algorithms in common use in network psychometrics were run on the same networks as robustness checks: a signed spin-glass algorithm [14], which preserves the sign of each edge, and Louvain [15] and Leiden [16], which optimize modularity. These three algorithms are reported in the Results only as checks on whether the Walktrap solution depends on the detection rule.

Bootstrap procedures

The point estimates under both principal specifications, Walktrap community detection, and the two 1,000-resample bootstraps constituted the main analyses; the remaining procedures were sensitivity or post hoc diagnostic checks. Sensitivity analyses varied the community-detection algorithm and the regularization strength (γ = 0, 0.25, and 0.50) under both correlation specifications, and, for the rank-based specification, additionally examined a nonparanormal-transformation network [17]; their full specification is given in Appendix B. For each specification, 1,000 nonparametric bootstrap resamples were drawn, with the network and communities re-estimated in each resample. We recorded how often all eight adversely framed items shared one community, how often that community contained no other items, and how many communities were found. Because the eight-item set is fixed by the scoring scheme rather than read off the network, these criteria need no matching of community labels across resamples. A post hoc diagnostic analysis of 300 additional polychoric resamples, specified in Appendix B, was run after the primary bootstrap to describe the partition patterns underlying the lower polychoric reproduction rate; it was not used to define or test the primary outcomes. Networks estimated under the two specifications were compared using edge-weight correlations and community agreement (the adjusted Rand index (ARI)) [18]. The ARI takes the value 1 when two partitions are identical and approximately 0 when their agreement is no better than chance. All 1,000 resamples per specification yielded an estimable network and community partition; the point-estimate polychoric matrix in the primary sample was positive definite (minimum eigenvalue = .055) and required no smoothing (Appendix B). Data were prepared in IBM SPSS Statistics software (IBM Corporation, Armonk, NY, USA); analyses used R: qgraph [19], bootnet [20], igraph, and psych.

No null hypothesis significance test was performed, and no p-value is reported in any table, figure, or section of this article. The R version (The R Core Team, R Foundation for Statistical Computing, Vienna, Austria), the version of each package, and the random seeds used are recorded in the analysis code referred to above.

## Results

Sample

The sample characteristics are shown in Table 1. Item-level distributional statistics after reverse-coding are presented in Table 2.

**Table 1:** Characteristics of the primary analysis sample (N = 1,014). Note. Disability registration in Muntinlupa is based on the leading type of disability as judged by clinicians; M: mean; SD: standard deviation. Age is expressed in years; all other entries are counts with the corresponding percentage in parentheses, n (%). No participant is registered with multiple types. Percentages may not total 100 owing to rounding.

Characteristic	Value
Age, years, M (SD) (range)	39.96 (13.77) (18–80)
Gender, n (%)	-
Male	505 (49.8)
Female	507 (50.0)
Gender diverse	2 (0.2)
Registered type of disability, n (%)	-
Orthopedic	565 (55.7)
Mental/psychosocial	205 (20.2)
Communication	100 (9.9)
Visual	80 (7.9)
Learning	34 (3.4)
Intellectual	30 (3.0)
Employment, n (%)	-
Employed	300 (29.6)
Not employed	714 (70.4)

Item-level response distributions were examined after reverse-coding, because the distributional properties of individual items bear on the correlation specifications used below. The corresponding descriptive statistics are presented in Table 2.

**Table 2:** Item distributional statistics after reverse-coding. M: mean; SD: standard deviation. All statistics were calculated after reverse-coding; skewness and excess kurtosis are moment-based (excess kurtosis = 0 under normality). Percentages at minimum and maximum indicate responses scored 0 and 4, respectively. Asterisks identify the reverse-keyed, adversely framed items. The minimum response-category cell count across items was 17. There were no missing Human Rights-Based Well-Being Checklist (HRWC) responses. M and SD are expressed in scale points on the 0–4 item response scale, and the two percentage columns are percentages of the 1,014 respondents.

Item	M	SD	Skewness	Excess kurtosis	Percentage at minimum (%)	Percentage at maximum (%)
1 *	3.36	1.10	−1.46	0.79	1.7	69.7
2 *	3.31	1.17	−1.61	1.44	5.2	67.7
3 *	3.09	1.22	−1.10	0.03	5.0	56.6
4 *	3.45	1.12	−1.97	2.59	4.5	76.7
5	2.39	1.43	−0.44	−1.11	16.8	29.8
6	2.12	1.44	−0.16	−1.28	21.1	23.5
7	2.06	1.42	−0.11	−1.23	21.5	21.1
8	2.33	1.33	−0.37	−0.97	14.0	24.1
9	2.32	1.42	−0.38	−1.14	17.4	26.8
10	2.45	1.23	−0.41	−0.69	9.2	24.7
11	2.01	1.37	−0.08	−1.19	20.4	17.5
12 *	2.51	1.33	−0.38	−1.03	9.3	33.1
13	2.06	1.36	−0.13	−1.13	19.1	18.1
14 *	2.61	1.36	−0.46	−1.05	9.3	39.7
15	2.24	1.44	−0.30	−1.22	19.4	25.7
16	1.99	1.45	−0.08	−1.32	24.7	19.7
17 *	3.12	1.32	−1.24	0.13	7.7	62.7
18 *	3.18	1.26	−1.31	0.38	6.1	64.1
19	2.41	1.42	−0.47	−1.07	16.3	29.2
20	2.54	1.45	−0.63	−0.96	16.8	35.3
21	2.94	1.23	−1.03	0.06	7.1	44.0
22	2.55	1.32	−0.58	−0.78	11.5	30.9
23	2.55	1.41	−0.61	−0.91	14.8	34.6
24	2.56	1.53	−0.66	−1.07	19.7	39.3
25	2.62	1.48	−0.71	−0.93	17.3	40.5
26	3.10	1.15	−1.20	0.56	5.1	51.5
27	2.73	1.22	−0.67	−0.46	7.1	35.3
28	2.42	1.38	−0.46	−1.02	14.4	28.4
29	2.66	1.20	−0.62	−0.45	7.1	30.6
30	2.60	1.19	−0.54	−0.52	7.2	28.3
31	2.55	1.20	−0.54	−0.54	8.1	25.4
32	2.19	1.43	−0.26	−1.23	20.1	23.9
33	2.38	1.48	−0.44	−1.20	19.1	31.1
34	2.83	1.22	−0.83	−0.24	6.9	39.3
35	3.05	1.14	−1.08	0.29	4.4	48.1
36	2.55	1.33	−0.52	−0.88	10.8	32.6
37	2.23	1.40	−0.24	−1.17	17.2	25.1
38	2.33	1.32	−0.27	−1.02	12.3	25.5
39	2.89	1.25	−0.94	−0.14	7.8	43.3

Network structure and reproducibility

The Spearman network contained 289 nonzero edges among the 39 items (density 39.0%; Figure 1) and five communities. The eight adversely framed items formed a single community, and the positively worded items were split into four. Within that community, 27 of the 28 possible partial correlations were positive and nonzero, and the remaining pair was zero (no edge). The polychoric network contained 290 edges (density 39.1%) and six communities: the adversely framed items were divided into Items 1-4 and Items 12, 14, 17, and 18 along the nearly identical stems of Items 1-4 (between-specification agreement: ARI = .86; edge-weight correlation r = .98). Within the eight items, no negative edges were present under either specification. Each stem-defined subgroup was fully positively connected internally under both specifications (six of six pairs). Of the 16 pairs linking the two subgroups, 15 were nonzero and positive under Spearman estimation, whereas nine were nonzero and positive under polychoric estimation.

**Figure 1:**
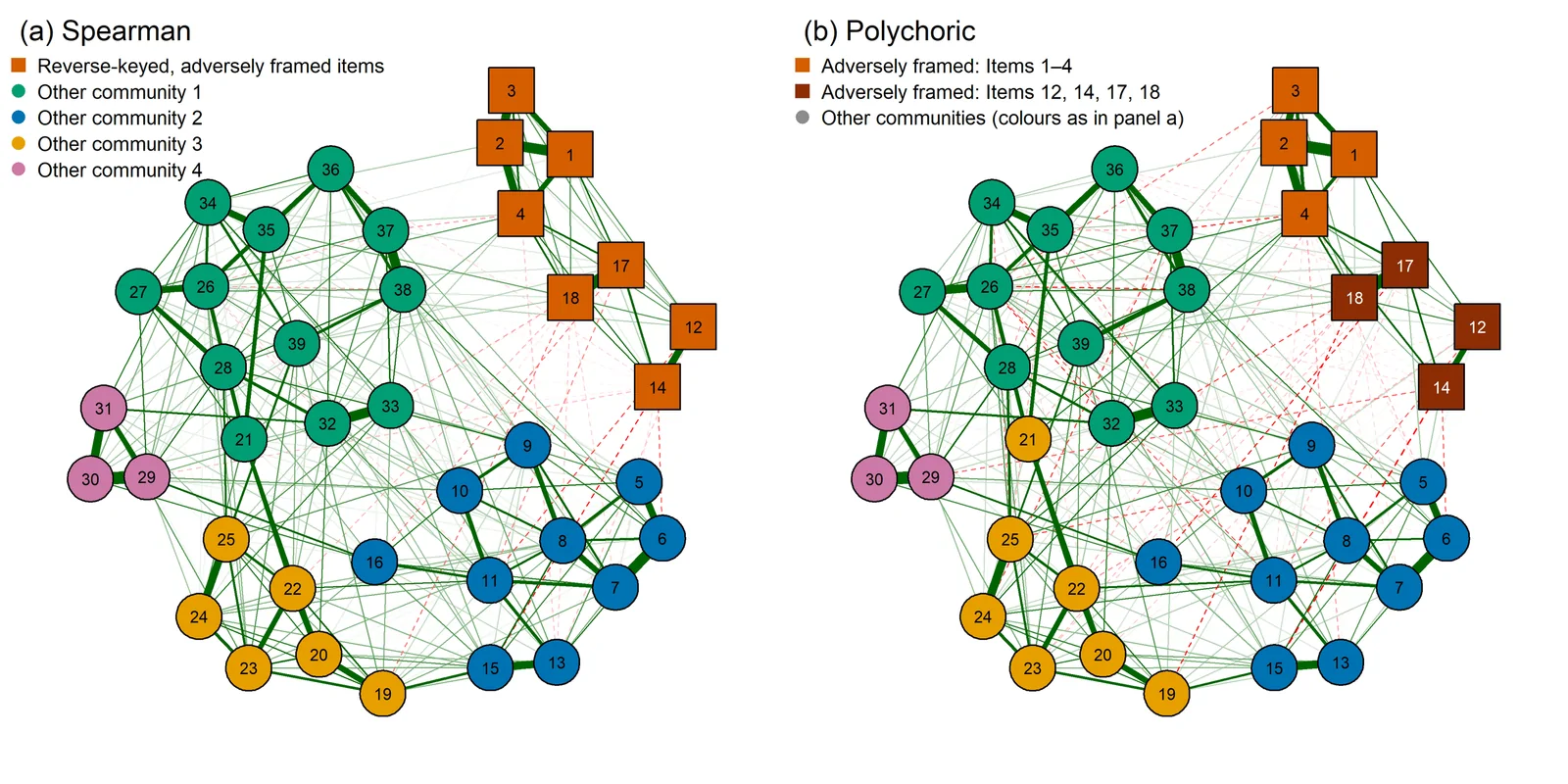
HRWC item networks (regularized partial correlations, N = 1,014) HRWC: Human Rights-Based Well-Being Checklist; EBICglasso: graphical lasso with extended-Bayesian-information-criterion model selection. (a) Spearman specification (289 edges, five communities); (b) polychoric specification (290 edges, six communities), in which the adversely framed items are divided into Items 1–4 and Items 12, 14, 17, and 18 under Walktrap detection. Node placement is identical in both panels (layout estimated from the Spearman network). The community colors show the Walktrap partitions. Node color indicates the network community; node shape indicates keying (squares, reverse-keyed, adversely framed; circles, positively worded). Edge color indicates the sign (solid/green, positive; dashed/red, negative); edge thickness is proportional to the absolute partial correlation. Layout: Fruchterman-Reingold ("spring"), fixed random seed (20260721); node placement is heuristic and does not represent statistical distance. The communities are descriptive groupings, not subscales; "Other communities 1–4" are arbitrary identifiers.

Edges linking the two sets were of mixed sign and, as conditional associations after reverse scoring, were not interpreted substantively. The edge-sign composition of the Spearman network is summarized in Table 3.

**Table 3:** Edge-sign composition in the Spearman network. N = 1,014. Edge signs represent conditional partial associations after reverse-coding and were not interpreted as simple substantive associations. Community detection used absolute edge weights. All nonzero edges within the adversely framed community were positive; therefore, this community was not produced solely by converting negative edge weights to absolute values. Entries are counts of edges with the corresponding percentage of the edge set in the same row.

Edge set	Non-zero edges	Negative	Percentage negative (%)
All item pairs	289	31	10.7
Within adversely framed community	27	0	0.0
Adversely framed × positively worded	41	25	61.0
Within positively worded items	221	6	2.7

Under Spearman estimation, the exclusive eight-item community was retained in the nonparanormal network, under the three alternative detection algorithms described in the Methods (Louvain, Leiden, and signed spin-glass detection; ARI vs. Walktrap .79-.84), and at γ = 0, 0.25, and 0.50 (identical membership; Table 4). Under polychoric estimation, the division of the eight items depended on the community-detection algorithm. Walktrap produced the two-group solution described above, whereas Louvain, Leiden, and signed spin-glass each placed all eight items in a single exclusive community (five, five, and six communities, respectively; ARI vs. Walktrap .79-.93). The two-group solution was unchanged at γ = 0, 0.25, and 0.50 (identical membership). The point-estimate community assignments across alternative detection algorithms and regularization settings are summarized in Table 4. In the Spearman Walktrap bootstrap, the eight items formed an exclusive community in 885 of the 1,000 resamples (88.5%), and five communities in 698 resamples (69.8%; range 4-9).

**Table 4:** Point-estimate community membership across detection algorithms and regularization settings. N = 1,014. ARI: adjusted Rand index; EBICglasso: graphical lasso with extended-Bayesian-information-criterion model selection; γ: EBICglasso tuning parameter. "All eight together" indicates that the eight adversely framed items fell in one community; "Exclusive" indicates that this community contained no positively worded items; "Shared-stem split" indicates the partition of Items 1–4 versus Items 12, 14, 17, and 18. Louvain, Leiden, and signed spin-glass were applied to the γ = 0.50 networks. ARI values were calculated against the Walktrap partition for the same correlation specification, with the γ = 0.50 Walktrap partition serving as the reference. Walktrap, Louvain, and Leiden used absolute edge weights, whereas signed spin-glass retained edge signs. No positively worded item joined the adversely framed items under any of these point-estimate conditions.

Network	Condition	No. of communities	All eight together	Exclusive	Shared-stem split	ARI vs reference
Spearman	Walktrap, γ = 0.50 (reference; 289 edges)	5	Yes	Yes	No	1.00
Spearman	Louvain	6	Yes	Yes	No	.84
Spearman	Leiden	6	Yes	Yes	No	.84
Spearman	Signed spin-glass	6	Yes	Yes	No	.79
Spearman	Walktrap, γ = 0 (306 edges)	5	Yes	Yes	No	1.00
Spearman	Walktrap, γ = 0.25 (306 edges)	5	Yes	Yes	No	1.00
Polychoric	Walktrap, γ = 0.50 (reference; 290 edges)	6	No	No	Yes	1.00
Polychoric	Louvain	5	Yes	Yes	No	.88
Polychoric	Leiden	5	Yes	Yes	No	.93
Polychoric	Signed spin-glass	6	Yes	Yes	No	.79
Polychoric	Walktrap, γ = 0 (442 edges)	6	No	No	Yes	1.00
Polychoric	Walktrap, γ = 0.25 (290 edges)	6	No	No	Yes	1.00

The stability of the eight-item community was then examined across the bootstrap resamples under each correlation specification. The reproduction rates are summarized in Table 5.

**Table 5:** Bootstrap reproduction of the eight-item community. N = 1,014; 1,000 nonparametric resamples per specification. Community detection used Walktrap in every resample. “Exclusive eight-item community” indicates that all eight adversely framed items formed one community containing no positively worded items. The criteria were defined by the composition of the eight-item set identified from the instrument’s scoring scheme and therefore required no matching of community labels across resamples. Percentages are conditional on Walktrap detection. No estimation failures occurred. Entries are percentages of those resamples, with the range of the number of communities across them.

Specification	All eight items in one community (%)	Exclusive eight-item community (%)	Most frequent number of communities (%)	Range of communities
Spearman	88.5	88.5	5 (69.8)	4–9
Polychoric	35.2	33.3	6 (53.2)	4–8

In the polychoric Walktrap bootstrap, all eight items remained in one community in 352 of the 1,000 resamples (35.2%) and formed an exclusive community in 333 resamples (33.3%); six communities were most common (532 resamples, 53.2%), followed by five (384 resamples, 38.4%). In the post hoc diagnostic resamples, the cluster divided in 191 of the 300 resamples (63.7%), almost always into two subgroupings (189 of the 191 divided resamples, 99%), most often along the shared stem (156 of 300 resamples, 52%). In 50 of the 300 diagnostic resamples (16.7%), a positively worded item shared a community with adversely framed items. A seed-exact audit revealed that the raw polychoric matrix was not positive definite and was smoothed automatically within estimation in 11 of 1,000 compositions and seven of 300 diagnostic resamples. The partition patterns underlying the lower polychoric reproduction rate are summarized in Table 6.

**Table 6:** Partition patterns of the eight adversely framed items in the polychoric diagnostic resamples. N = 1,014; 300 post hoc diagnostic resamples. Community detection used walktrap. These diagnostic resamples were separate from the 1,000-resample primary bootstrap summarized in Table 5. The diagnostic analysis was conducted after the primary bootstrap to describe the partition patterns underlying the lower polychoric reproduction rate and was not used to define or test the primary outcomes. In 50 resamples (16.7%), at least one positively worded item joined a community containing adversely framed items; this was an overlapping diagnostic characteristic rather than a mutually exclusive partition category. Raw-matrix smoothing was required in seven resamples (2.3%). Entries are counts of resamples with the corresponding percentage of the 300 diagnostic resamples.

Partition	Resamples	% of 300
Intact ({1, 2, 3, 4, 12, 14, 17, 18})	109	36.3
Shared-stem division ({1–4} vs {12, 14, 17, 18})	156	52.0
Other two-subgroup divisions	33	11.0
Three or more sub-groupings	2	0.7

## Discussion

Across all the examined point-estimate specifications, the eight reverse-keyed, adversely framed items were separated from the positively worded items, and no positively worded item joined them under any of these conditions. The internal grouping, however, was not stable. The eight items formed either one community or two subgroups depending on the correlation specification, the community-detection algorithm, and the bootstrap resample. The observed communities, therefore, did not provide a sufficiently stable basis for constructing subscales or removing items. Under rank-based estimation, the exclusive community appeared in the point estimate and was retained across the examined Spearman specifications: alternative algorithms, regularization strengths, and the nonparanormal transformation. It reproduced in 885 of the 1,000 bootstrap resamples (88.5%). Under ordinal estimation, the Walktrap point estimate divided the eight items into two groups along the shared stem of Items 1-4, a division that three alternative detection algorithms did not reproduce, and the exclusive community was reproduced in a third of the resamples. The polychoric sensitivity analyses help localize the source of this variation. Changing the regularization parameter did not alter the Walktrap partition, whereas changing the detection algorithm did. The one-versus-two-community distinction was therefore more sensitive to the detection rule than to regularization strength, while the broader separation of the adversely framed items from the positively worded items was retained. This divergence is itself informative: a community clear under one defensible specification need not have an invariant composition under another, even for the same items and respondents. Bootstrap percentages describe the stability of these classifications under resampling from the present sample; they are not probabilities that a true community exists in the population.

Sources of the observed grouping

This distinction concerns framing, not whether an item addresses rights or rights violations: all items concern rights conditions, and low scores on positively worded items can also indicate less favorable reported rights-related conditions. The observed grouping should not be interpreted as evidence of a wording effect alone [6]. Substantive rights-violation content, keying direction, negative valence, the near-identical stem of Items 1-4, item position, and response distributions coincide in these items; the present design cannot separate these sources. Ceiling concentration varied across the adversely framed items, yet the observed division followed the shared stem rather than separating the most extreme-ceiling items (Table 2); the ceiling alone did not affect the composition, although broader distributional influences could not be excluded. For an instrument whose items were selected to cover heterogeneous CRPD-related content rather than to maximize expected interitem covariance, the separation of communities is not necessarily a defect.

These findings can be placed alongside the studies of reverse-keyed items cited above. Those studies reported that reverse-keyed items group together and attributed the grouping to careless responding [6], to wording effects that are not resolved by modeling them explicitly [7], and to the influence of item keying on the recovered factor structure [8]. The present results are consistent with them in that a keying-related grouping was again observed. Two features of the present case are not addressed in that literature. First, the instruments examined there are reflective scales, in which such a grouping is treated as a nuisance to be modeled away or removed; here the grouped items describe distinct CRPD-related conditions that are not interchangeable, so removing or separating them would change what the instrument covers. Second, the grouping was not invariant across defensible specifications in these data: whether the eight items formed one community or two subgroups depended on the correlation specification and the detection algorithm. Both features bear on what may be inferred from a keying-related community in a content-coverage instrument, and they underlie the practical implications set out below.

Practical and methodological implications

This study has three practical and methodological implications. First, item-level profiles and the existing unweighted total serve different descriptive purposes. Item-level profiles preserve information on the distinct CRPD-related conditions represented by individual items. Under the existing scoring convention [2], the unweighted total may be reported alongside these profiles as a concise summary across all 39 responses, rather than as a latent trait score. The validity of that scoring convention, and the utility of the HRWC for service planning, are questions for separate study. Second, the findings do not support data-driven changes to the scoring structure. The observed communities did not provide a stable basis for replacing the existing total with empirically derived subscales, removing items, or changing item weights. Treating the network communities as reflective subscales, or dropping or downweighting items because of their covariance pattern, could raise apparent homogeneity at the cost of coverage of rights the CRPD addresses explicitly: discrimination, threats to life, unlawful detention, and violence. Identifying wording-related structures does not by itself reveal that alternative scoring yields less-contaminated person scores [7]. Third, the results identify priorities for future instrument development, including shared item stems, item order, matched alternative phrasings, and administration procedures; experimental studies could, for example, compare mixed and blocked administration of positively and negatively worded content [8].

These implications extend beyond the HRWC: in other content-coverage indices, a distinct community may reflect substantive co-occurrence, a shared item format, response distributions, or their combination. Such communities can be assessed in sequence: content or item features first, robustness across specifications second, and scoring implications only thereafter. Here, the first two steps identified a noteworthy response-structure feature, whereas the third provided no support for changing the scoring or item content.

Limitations

This is a single registry-based sample from one city, drawn from a parent survey in which 1,024 of 3,000 approached registrants participated (34.1%), and selection related to functional or communication difficulties cannot be excluded. The language used with each respondent, any verbal assistance or rephrasing by the health workers, and proxy involvement were not recorded; therefore, the contribution of language and administration procedures to the observed clustering could not be assessed. Underrepresentation of persons reporting the most severe adverse rights-related experiences could alter edge strengths and community boundaries; the network describes the participating registry sample. Most of the adversely framed items showed strong ceiling effects (up to 76.7% at maximum) and a marked negative skew (to -1.97), which may have contributed to the polychoric instability observed under resampling and are inseparable from the keying direction (Table 2). Bootstrap community-reproduction estimates were conditional on Walktrap detection and should not be interpreted as algorithm-independent stability estimates. The networks were relatively dense when γ = 0.50; thus, caution is warranted when the smallest edges are used. No analysis plan was preregistered, and all analyses were exploratory. The decision to focus on the eight adversely framed items followed exploratory inspection of the estimated networks; although the set itself was defined from the scoring scheme rather than from the network results, its reproducibility analysis was post hoc and requires confirmation in independent data. The CRPD-article mapping in Appendix A was compiled with instrument developers who are coauthors and was not independently validated; it is provided for interpretive context and was not analyzed as an outcome. All analyses are cross-sectional descriptions of response structure; causality, invariance, retest reliability, and responsiveness are not addressed.

## Conclusions

The adversely framed items were broadly separated from the positively worded items across the examined point-estimate specifications, but their exact internal grouping was not invariant. Under ordinal estimation, Walktrap divided them into two groups along the shared stem of Items 1-4, whereas the three alternative algorithms kept all eight in one exclusive community. Item-level profiles and the existing unweighted total serve different descriptive functions: the former preserve information on distinct CRPD-related conditions, whereas the latter, to which all 39 responses contribute equally, can provide a concise summary across all 39 responses. The observed communities provided no stable basis for constructing new subscales, removing items, or changing item weights, and substantive content could not be separated from keying, stems, position, and response distributions. Diagnostic use and the interpretation of change remain unvalidated. More broadly, data-derived communities in content-coverage instruments are best treated diagnostically and checked across specifications before scoring or content is modified.

## Appendices

Appendix A

HRWC Items, CRPD-Article Mapping, and Reverse-Keying

**Table 7:** Human Rights-based Well-being Checklist (HRWC) items, Convention on the Rights of Persons with Disabilities (CRPD) -article mapping, and reverse-keying. Note: Reverse-keyed items (1–4, 12, 14, 17, 18) are the adversely framed items discussed in the main text. This appendix lists item content and article mapping only; it contains no measured values, so no statistical test was applied and no p-value is reported. In the printed bilingual questionnaire, the near-identical stem of Items 1–4 appears in both administered languages; the Filipino wording of Items 1–4 shares the stem "Ako ay nakakaranas ng diskriminasyon ..." (reproduced from the study questionnaire).

No.	HRWC item	CRPD article(s)	Article title	Reverse-keyed
1	I am suffering from inequality & discrimination due to my sex/gender.	Arts. 5, 6	Equality and Non-Discrimination; Women with Disabilities	Yes
2	I am suffering from inequality & discrimination due to my age.	Arts. 5, 7	Equality and Non-Discrimination; Children with Disabilities	Yes
3	I am suffering from inequality & discrimination due to my disability.	Art. 5	Equality and Non-Discrimination	Yes
4	I am suffering from inequality & discrimination due to my race or ethnicity.	Art. 5	Equality and Non-Discrimination	Yes
5	I am respected as a capable person.	Art. 8	Awareness-Raising	No
6	I can move around in this city without much barriers.	Arts. 9, 20	Accessibility; Personal Mobility	No
7	I can use necessary transportation to participate in social life.	Arts. 9, 20	Accessibility; Personal Mobility	No
8	I can access necessary information to participate in social life.	Art. 9	Accessibility	No
9	I can access internet and other new technologies.	Art. 9	Accessibility	No
10	I can ask for support from others when needed.	Art. 9	Accessibility	No
11	I can participate in decision-making related to my community and nation if I want.	Art. 4	General Obligations	No
12	I feel threats to my life in my daily life.	Art. 10	Right to Life	Yes
13	I think I can protect my minimum safety when disasters happen in this city.	Art. 11	Situations of Risk and Humanitarian Emergencies	No
14	My legal decision-making will be obstructed by others.	Art. 12	Equal Recognition Before the Law	Yes
15	I can protect my money and assets without interference from others.	Art. 12	Equal Recognition Before the Law	No
16	I can access legal services such as the court when needed.	Art. 13	Access to Justice	No
17	I can be detained unlawfully.	Arts. 14, 15	Liberty and Security of Person; Freedom from Torture or Cruel, Inhuman or Degrading Treatment or Punishment	Yes
18	I can be subject to physical or sexual violence.	Art. 16	Freedom from Exploitation, Violence and Abuse	Yes
19	I can make decisions on my body and mind without interference from others.	Art. 17	Protecting the Integrity of the Person	No
20	I can decide where to live.	Art. 18	Liberty of Movement and Nationality	No
21	My place of living is OK.	Art. 19	Living Independently and Being Included in the Community	No
22	I can make my opinion heard when needed.	Art. 21	Freedom of Expression and Opinion, and Access to Information	No
23	I can keep my privacy when I wish to.	Art. 22	Respect for Privacy	No
24	I can participate in fair election.	Art. 29	Participation in Political and Public Life	No
25	I can marry and have a child without much interference from others, if I want.	Art. 23	Respect for Home and the Family	No
26	I can communicate with my family when I want to.	Art. 23	Respect for Home and the Family	No
27	I can communicate with my friends when I want to.	Arts. 19, 21	Living Independently and Being Included in the Community; Freedom of Expression and Opinion, and Access to Information	No
28	I can have/had quality education.	Art. 24	Education	No
29	I can receive necessary health services when I would have a physical health condition.	Art. 25	Health	No
30	I can receive necessary health services when I would have a mental health condition.	Art. 25	Health	No
31	I think I can receive necessary social services to live in community when needed.	Art. 26	Habilitation and Rehabilitation	No
32	I can choose my work if I want to.	Art. 27	Work and Employment	No
33	My work environment is/would be OK (If I'd work).	Art. 27	Work and Employment	No
34	My standard of living (food, clothing and housing) is adequate.	Art. 28	Adequate Standard of Living and Social Protection	No
35	I have access to clean water and toilet.	Art. 28	Adequate Standard of Living and Social Protection	No
36	I can access/enjoy culture and the arts (including music, films, theatres, museums, libraries, etc.)	Art. 30	Participation in Cultural Life, Recreation, Leisure and Sport	No
37	I can access/enjoy sports.	Art. 30	Participation in Cultural Life, Recreation, Leisure and Sport	No
38	I can enjoy tourism and leisure when I want to.	Art. 30	Participation in Cultural Life, Recreation, Leisure and Sport	No
39	I have freedom to keep my culture and religion.	Art. 30	Participation in Cultural Life, Recreation, Leisure and Sport	No

Appendix B

Sensitivity and Diagnostic Analyses

This appendix documents the procedures summarized in the Statistical analysis section. None of them was used to define or test the primary outcomes, which were the point estimates under the two principal correlation specifications, the Walktrap community detection, and the two 1,000-resample bootstraps.

Regularization settings: Under both correlation specifications, the EBICglasso tuning parameter was varied across γ = 0, 0.25, and 0.50, and the network and the Walktrap communities were re-estimated at each value. The resulting point-estimate community memberships are reported in Table 4. Membership was identical across the three settings within each specification: under Spearman estimation the eight adversely framed items formed one exclusive community at every setting, and under polychoric estimation the shared-stem division of Items 1-4 versus Items 12, 14, 17, and 18 was obtained at every setting. The number of estimated edges differed between settings and is given in Table 4.

Alternative community-detection algorithms: Louvain, Leiden, and the signed spin-glass algorithm were applied to the γ = 0.50 networks under both correlation specifications. Walktrap, Louvain, and Leiden used absolute edge weights, whereas the signed spin-glass algorithm retained edge signs. Agreement with the Walktrap partition for the same correlation specification was quantified with the ARI and is reported in Table 4.

Nonparanormal transformation: The nonparanormal check applies to rank-based estimation and was therefore run for the Spearman network only. The transformed network retained the exclusive eight-item community, and the edge weights of the transformed and untransformed Spearman networks correlated at .997.

Post hoc diagnostic resamples: For the polychoric specification, 300 additional bootstrap resamples were drawn after the primary bootstrap in order to describe how the eight adversely framed items were partitioned in resamples in which they did not form a single exclusive community. These 300 resamples were separate from the 1,000-resample primary bootstrap and were not used to define or test the primary outcomes. The resulting partition patterns are reported in Table 6.

Matrix smoothing audit: A seed-exact audit recorded how often the raw polychoric correlation matrix was not positive definite and was, therefore, smoothed automatically within estimation. Smoothing was applied in 11 of the 1,000 primary bootstrap compositions and in seven of the 300 diagnostic resamples. In the primary sample, the point-estimate polychoric matrix was positive definite (minimum eigenvalue = .055) and required no smoothing. All 1,000 resamples per specification yielded an estimable network and community partition, and no estimation failures occurred.
